# Domestic Activities Associated With a Decreased Risk of Cognitive Disorders: Results of the “Fréle” Cohort

**DOI:** 10.3389/fpubh.2020.602238

**Published:** 2020-12-09

**Authors:** Caroline Dupré, David Hupin, Luc Goethals, François Béland, Frédéric Roche, Thomas Célarier, Isabelle Carrière, Nathalie Barth, Bienvenu Bongue

**Affiliations:** ^1^Centre technique d'appui et de formation (CETAF), Saint-Etienne, France; ^2^Laboratory SNA-EPIS EA 4607, University Jean Monnet, Saint-Etienne, France; ^3^Chaire Santé des ainés et ingénierie de prévention, Saint-Etienne, France; ^4^Département de Gestion, d'évaluation et de politique de santé, École de santé publique, Université de Montréal, Montreal, QC, Canada; ^5^Institute Lady Davis, Jewish General Hospital, Montréal, QC, Canada; ^6^Service de gérontologie, Hôpital Nord, Centre Hospitalier Universitaire, Saint-Etienne, France; ^7^Univ. Montpellier, Inserm, PSNREC, Montpellier, France; ^8^Gérontopôle Auvergne Rhône-Alpes, Saint-Etienne, France

**Keywords:** physical activity, older people, cognitive decline, cohort, domestic

## Abstract

**Background:** Previous cohorts studied the association of various types of physical activities with the incidence of cognitive disorders. The objective of this work was to analyze the association of leisure, domestic and professional physical activities with mild and moderate cognitive disorders in older people living in the community.

**Methods:** We used retrospective data from the “FRéLE” (FRagilité: étude Longitudinale de ses Expressions) a longitudinal and observational study. Data collected included socio-demographic variables, lifestyle and health status. Cognitive disorders were assessed using the Montreal Cognitive Assessment (MoCA). Two cut-offs of MoCA were used to analyze mild and moderate cognitive disorders. Physical activity was assessed by the Physical Activity Scale for the Elderly (PASE) structured in three sections: leisure, household, and professional activities. Spline and logistic regression models were used to estimate the risk of cognitive disorders.

**Results:** At baseline, 428 participants (for study of mild disorders) and 1,271 participants (for study of moderate disorders) without cognitive disorders were included in the analysis. The mean ages were 74 and 78 years, respectively. After a 2-year follow-up, we found mild cognitive disorders in 154 participants (36%) and 71 cases of moderate cognitive disorders (5.6%). In multi-adjusted logistic models, domestic activities were associated with cognitive disorders, but not leisure and professional activities.

**Conclusion:** We found an inverse relation between domestic sub-score and cognitive disorders defined by MoCA < 18. With a specific questionnaire and quantitative information on the type of activities, this study contributed to the debate on the beneficial effects of physical activity on cognition.

## Introduction

The advancing age of a population leads to an increase in age-related pathologies, including neurodegenerative diseases. However, aging should not be synonymous with poor health, for age-related diseases can be avoided or delayed. The importance for older adults to carry out physical activities (PA) even during the ongoing COVID-19 pandemic is well-known. This practice is important because its health benefits are well established (1). A recent study found a positive impact of PA on myocardial infarction and stroke in adults aged more than 65 years (2). Physical activity is a non-drug prevention solution (3, 4). According to a recent meta-analysis, a minimum would reduce mortality by 20% compared to no practice of leisure-time PA (5).

The link between PA and cognitive decline is commonly discussed in literature. Although several studies suggest that it can improve cognitive function and reduce cognitive decline (6), the debate continues regarding the magnitude of its effects, the cognitive domains most affected, and the effects of different types. Systematic review of Cunningham (7), showed a reduction of the risk of cognitive decline (26% for moderate physical activity and 33% for high physical activity). But others meta-analyses (8, 9) conclude that physical activity wasn't associated with cognition. Sabia et al. (10), in a large cohort study, found no evidence of a neuroprotective effect of PA. Two hypotheses could explain this inconsistent result: the way the activities were assessed among the elderly and the tools used to assess the cognitive decline.

A few cohorts have studied the different types of PA, but did not investigate household activities (11). In a recent study carried out on the FRéLE data, Beland et al. (12) showed a link between cognition and mobility but did not investigate the types of these activities.

The objective of this investigation (or study) was to analyze the associations between leisure, domestic, and professional activities and the incidence of mild and moderate cognitive disorders in community-living older adults. The question was to find out if one type of physical activity had a different relation with cognition than the others types.

## Methods

### Study Design

We used data from the FRéLE longitudinal study. To understand the incidence of the occurrence of cognitive decline during the follow-up period and to take into account the degree of severity (low or moderate) of the disorder. We studied distinct subpopulations, excluding those who already had the condition at the baseline.

The FRéLE cohort (13) is a multi-site longitudinal and observational study with a two-year follow-up of community-dwelling participants aged 65 years or older. This involved recruiting 1,643 participants between 2010 and 2012 in three Regions: CSSS Saint-Laurent-Bordeaux-Cartierville at Montréal, CSSS “University Intitute of Geriatrics” at Sherbrooke and CSSS Des Érables at Victoriaville. The aim was to analyze the components of frail profiles and to determine their consequences on health. The FRéLE study was made up of three face-to-face interviews with a one-year interval and two telephone interviews. Participants were drawn at random from the list of elderly people registered in the Universal Health Insurance Regime of Quebec (RAMQ). The inclusion criteria were: living in a private household and not being hospitalized. Data collected included social and psycho-social aspects (social network, income, education, among others.), cognition tests, PA, mobility, chronic diseases (physical comorbidity, depression), physiological impairments (sight, hearing, functional limitations of the lower limbs), functional disabilities (ADL—Activities of Daily Living, DADL—Domestic Activities of Daily Living, incontinence) and perceived health.

### Cognition Assessment

Cognitive disorder was evaluated using the Montreal Cognitive Assessment (MoCA) screening tool (14). It is used for the detection of mild cognitive impairment that distinguishes individuals with this condition from those who are aging normally. It assesses the following cognitive spheres: attention, concentration, executive functions, memory, language, visual-constructive skills, abstraction skills, calculations and orientation. The maximum score is 30 points and the pathological threshold is <26/30. In general, a result between “27–30” is considered normal, “18–26” indicates the presence of mild cognitive impairment, “10–17” the presence of moderate cognitive impairment, and “ <10” the presence of severe cognitive impairment (15). With an inclusion score <27, MoCA therefore makes it possible to detect mild cognitive disorders (16) while <18 indicates moderate cognitive impairment. We used two cut-offs (27 and 18) to define mild and moderate disorders.

Participants with cognitive disorders at baseline were excluded from our two analyses. In the first analysis, cognitive disorder was defined with MoCA <27 (vs. no cognitive decline: MoCA ≥ 27), and in the second analysis, it was defined with MoCA <18(vs. no cognitive decline: MoCA ≥ 18).

### Physical Activity Assessment

The Physical Activity Scale for the Elderly (PASE) (17), a 12-item self-administered questionnaire designed to measure the amount of physical activity in individuals over the age of 65, was used for the assessment. It assesses the types of professional, domestic, and leisure activities (walking, recreation, exercise, housework, gardening, and caring for others). It uses the frequency, duration and intensity level of activities in the previous week and assigns a score ranging from 0 to 793, with higher scores indicating greater physical activity. Professional activity corresponds to work-related activities (carried out for a salary or voluntarily). In order to evaluate the different types of activity, we studied the three sub-scores separately.

### Covariates

The socio-demographic variables at baseline sex, age and educational level. Two measures of frailty were included using the Groningen Frailty Indicator (GFI) (18) and the Fried scale (19). The GFI determine level of frailty with fifteen items on four domains (physical, cognitive, social, and psychological): frailty is defined with a score ≥ 4. It was used in the analyses in quantitative so as to keep all the information on the frailty of the participant. The Fried scale permits to indicate if the population is no frailty, pre-frailty or frailty. Health status covariates at baseline included the presence of depression [Geriatric Depression Scale - GDS (20)], heart disease, stroke, arthritis and diabetes.

### Statistical Analyses

The use of two cut-offs led to the realization of two hands using the same statistical method. Logistic regression models were used to estimate the risk of cognitive disorders, while spline modeled the non-linear relationship between the PASE and cognitive disorders. Associations between variables at baseline and the incidence of cognitive disorders during the follow-up were first of all adjusted for sex, age and educational level (Model 1). Every significant covariate in this model was then included in a multi-adjusted model (Model 2). Odds Ratios (OR) were presented with their 95% Confidence Interval (95% CI). The splines allowed to model the relationship between the domestic and leisure sub-cores of PASE and cognitive disorders by polynomials. The distribution of professional activities did not permit to use the splines. The best model was obtained by varying the degree of the polynomial and the choice of knots. In our case, we tested degrees 2 and 3, the higher degree providing no additional information. The knots tested were percentiles (deciles, quintiles, quartiles, tertiles) and were recommended by Harrel (21). In the case of polynomials of degree 3, we used restricted cubic splines. Indeed, splines are sensitive to the values observed in the first and last intervals, and the restricted cubic splines make it possible to add the constraint of being linear over the two extreme intervals. The best model was chosen with the lowest Akaike Information Criterion (AIC). The two median knots were used to transform the two sub-scores into classes. Statistical analyses were performed using the SAS software (version 9.4; SAS Institute Inc., Cary, NC).

## Results

### Physical Activities and Mild Disorder (MoCA < 27)

Of the 1,643 participants, 1,124 (68.4%) were not included because they were considered as having mild cognitive disorder (MoCA <27) at the baseline. Of the 453 participants left, 25 were excluded because they were lost to follow-up. Among the 428 participants finally included, 36% presented mild cognitive disorder during the follow-up with the MoCA test (**Figure 2**).

The mean age was 74 years, and 60.5% of the participants were women. Some 24.8% were in a state of frailty according to the GFI, and 9.6% according to the Fried scale (35.8% in pre-frailty). Depressive symptoms (GDS) were present in 14% of the sample population. Furthermore, 11% had myocardial infarction and 11.7% had diabetes (Table 1).

**Table 1:** Baseline characteristics and cognitive disorders incidence during follow-up.

	**Without cognitive disorders (*n* = 274)**	**Withcognitive disorders (*n* = 154)**	OR (95% CI)^*^	***p***
	***n* (%)**	***n* (%)**		
**Cognitive disorders at the end of follow-up (MoCA** ** <27)**				
Sex, female	177 (64.6)	82 (53.3)	0.54 (0.35–0.84)	0.006
GFI - Frailty	55 (20.1)	51 (33.1)	1.81 (1.12–2.93)	0.016
FRIED				0.204
Pre-frail	86 (31.4)	67 (43.5)	1.15 (0.53–2.49)	
Frail	22 (8.0)	19 (12.3)	1.51 (0.96–2.40)	
Cardiovascular disease	18 (6.6)	125 (81.2)	2.52 (1.28–4.98)	0.008
Diabetes				
Depressive symptoms (GDS)	27 (9.8)	33 (21.4)	2.36 (1.29–4.35)	0.017
	**Mean (sd)**	**Mean (sd)**		
Educational level	13.2 (4.2)	11.6 (4.4)	0.92 (0.87–0.97)	0.001
Age	73.4 (6.7)	77.7 (7.5)	1.08 (1.05–1.11)	< .0001
	**Without cognitive disorders (*****n*** **=** **1,200)**	**With cognitive disorders (*****n*** **=** **71)**	OR (95% CI)^*^	***p***
	***n*** **(%)**	***n*** **(%)**		
**Cognitive disorders at the end of follow-up (MoCA** ** <18)**				
Sex, female	611 (50.9)	35 (49.3)	0.87 (0.53–1.43)	0.578
Depressive symptoms (GDS)	2.6 (2.5)	3.7 (2.2)	1.09 (1.00–1.19)	0.060
GFI - Frailty	348 (29.0)	36 (50.7)	1.76 (1.06–2.93)	0.029
FRIED				0.054
Pre-frail	512 (42.7)	33 (46.5)	2.53 (1.19–5.37)	
Frail	150 (12.5)	24 (33.8)	1.60 (0.83–3.12)	
Arthritis	522 (43.5)	45 (63.4)	2.02 (1.19–3.41)	0.009
Stroke	93 (7.8)	12 (16.9)	1.96 (0.98–3.89)	0.056
Diabetes	202 (16.9)	18 (25.4)	1.46 (0.81–2.61)	0.208
	**Mean (sd)**	**Mean (sd)**		
Educational level	11.3 (4.6)	7.8 (3.7)	0.84 (0.79–0.90)	<0.0001
Age	77.3 (7.6)	83.6 (6.1)	1.10 (1.06–1.15)	<0.0001

* adjusted for age, gender and educational level.

*OR, Odds ratio; CI, Confidence Interval*.

**Figure 1:**
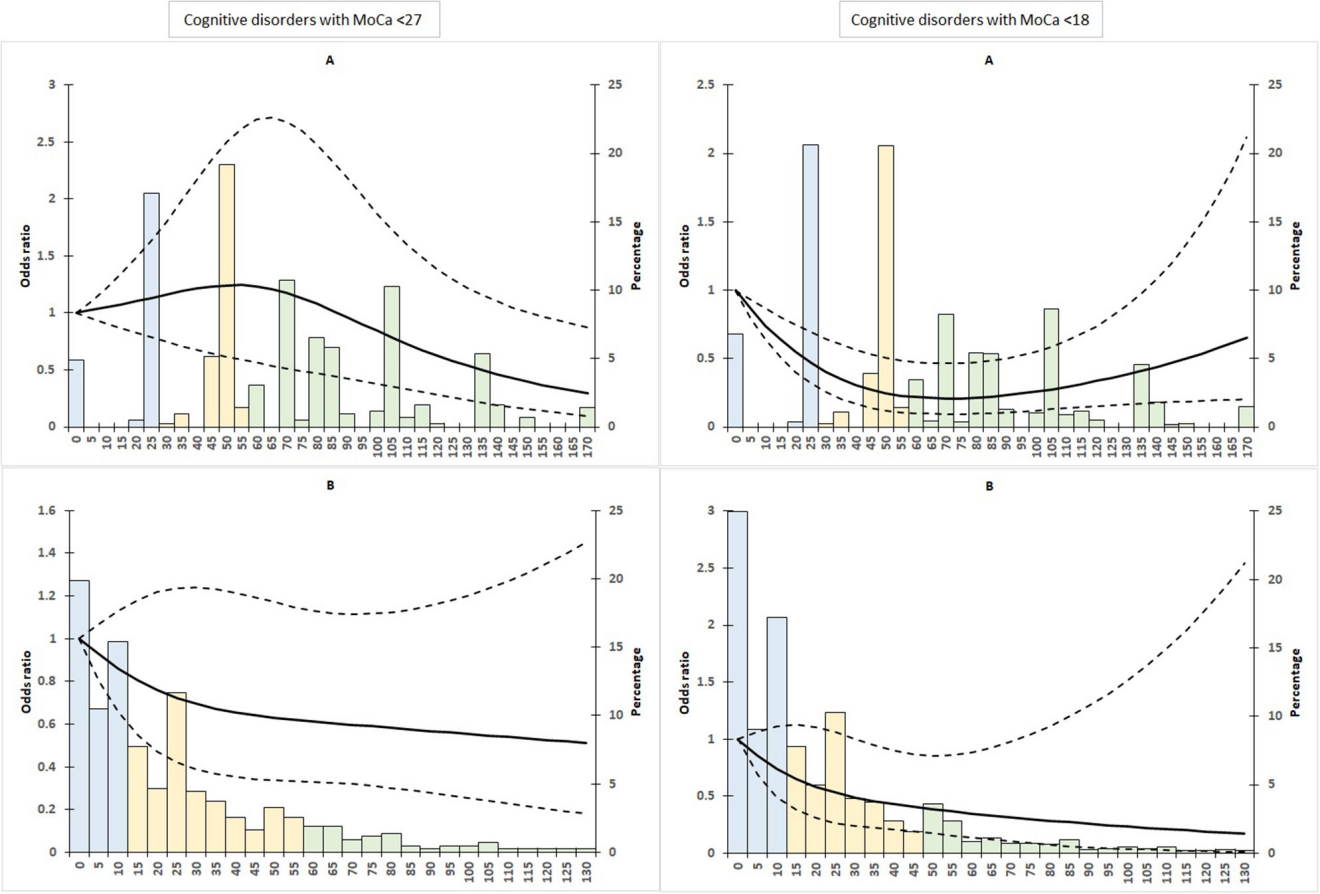
Adjusted (age, gender, years of education) spline analyses of cognitive decline risk (MoCA) by domestic activities **(A)** and leisure activities **(B)**. Dashed lines represent 95% confidence intervals. The score distribution is represented by the histogram in the background with the blue bars for the reference class, the yellow bars for the moderate activity class and the green bars for the high activity class.

The median sub-score (IQR) was 60 (45–86) for domestic activities and 15 (5–33.4) for leisure activities. The professional sub-score was zero in 82.2% of the participants. The best-fit model was obtained with the 10-50-90 percentiles for domestic and leisure activities. The splines were not significant for the domestic (*p* = 0.0532) and the leisure activity scores (*p*-value = 0.2683) (Figure 1). Given the distribution of professional activities, no spline was realized.

The domestic and leisure PASE sub-scores were transformed into three classes from the knot splines: [0–25], ]25–60[, >=60 for the first, and [0–15[, [15–60[, >=60 for the second. Taking into account the proportion of zeros from the professional score, only two classes were created: professional vs. no activities. The minimally adjusted logistic regression (Model 1) and the multi-adjusted model (Model 2) showed no significant results (Table 2).

**Table 2:** Association between baseline physical activity and risk of cognitive disorders.

**Cognitive disorders at the end of follow-up (MoCA** **<** **27)**				
	**Model 1: Minimally adjusted**^**a**^		**Model 2: Multi-adjusted**^**b**^	
	***n*****/*****N*** **=** **154/428**		***n*****/*****N*** **=** **154/427**	
	**OR (95% CI)**	***p***	**OR (95% CI)**	***p***
**Domestic activities**				
[0–25]	–	–	–	–
]25–60[	1.04 [0.57–1.89]	0.522	1.38 [0.73–2.61]	0.216
>=60	0.78 [0.45–1.37]	0.249	1.02 [0.56–1.85]	0.536
**Leisure activities**				
[0–15[	–	–	–	–
[15–60[	0.87 [0.56–1.37]	0.341	1.03 [0.65–1.66]	0.248
>=60	0.46 [0.21–1.04]	0.076	0.57 [0.25–1.32]	0.159
**Professionnal activities**				
No	–	–	–	–
Yes	1.28 [0.73–2.24]	0.388	1.41 [0.80–2.50]	0.237
**Cognitive disorders at the end of follow-up (MoCA** **<** **18)**				
	**Model 1: Minimally adjusted**^**a**^		**Model 2: Multi-adjusted**^**c**^	
	***n*****/*****N*** **=** **71/1,271**		***n*****/*****N*** **=** **71/1,269**	
	**OR (95% CI)**	***p***	**OR (95% CI)**	***p***
**Domestic activities**				
[0–25]	–	–	–	–
]25–50[	0.27 [0.12–0.57]	0.006	0.31 [0.14–0.67]	0.008
>=60	0.55 [0.31–0.99]	0.830	0.71 [0.38–1.32]	0.447
**Leisure activities**				
[0–15[	–	–	–	–
[15–50[	0.53 [0.28–1.00]	0.857	0.62 [0.32–1.21]	0.827
>=50	0.32 [0.10–1.09]	0.193	0.46 [0.13–1.59]	0.392
**Professionnal activities**				
No	–	–	–	–
Yes	1.14 [0.49–2.66]	0.755	1.41 [0.60–3.33]	0.437

n/N = number of cases/total.

OR, Odd Ratio; CI, Confidence Interval.

a adjusted for age, gender and years of education.

b adjusted for age, gender, years of education, depression (GDS), cardiovascular diseases, diabetes, frailty (GFI).

c *adjusted for age, gender, years of education, depression (GDS), arthritis, stroke, diabetes, frailty (GFI)*.

### Physical Activities and Moderate Disorder (MoCA < 18)

**Figure 2:**
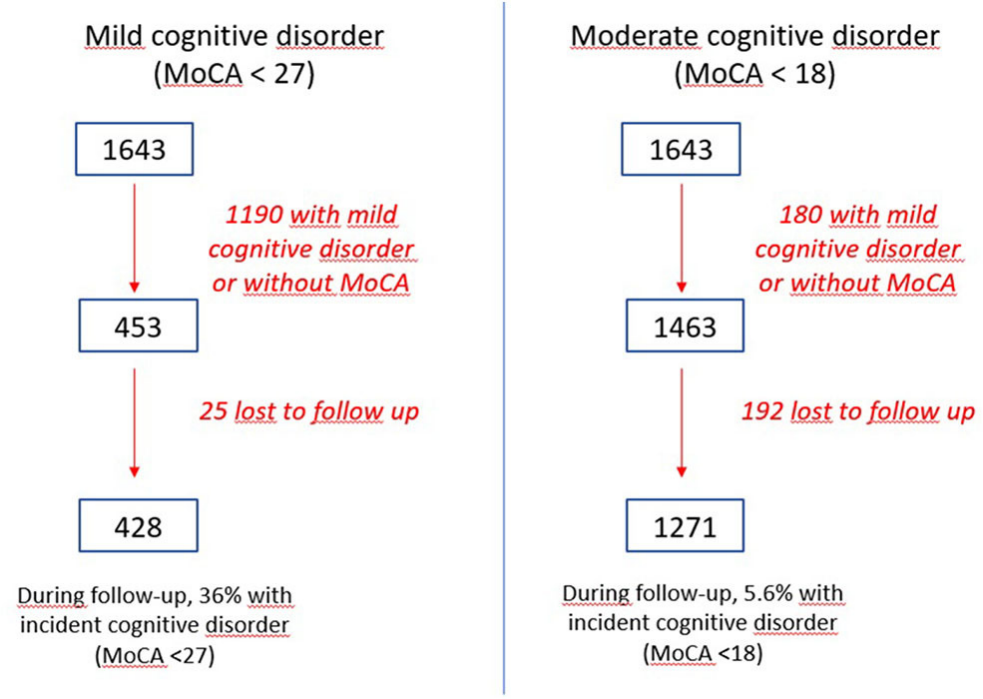
Flow chart of the two analyses.

Using the cut-off of <18 with the MoCA test on the 1,643 participants at baseline, 114 were excluded because they were considered as presenting moderate cognitive disorders, while 192 were excluded due to no participation in follow-up interviews. Of the 1,271 participants included, 5.6% presented moderate cognitive disorder after a 2-year follow-up (Figure 2).

Women represented 50.8% of the participants, and the mean age was 77.6 years. Some 30.2% were in a state of frailty according to the GFI, 13.7% according to the Fried scale (42.9% pre-frailty), and 19% had depressive symptoms according to the GDS. Furthermore, 13.8% had myocardial infarction and 17.3% had diabetes (Table 1).

For domestic activities, the median sub-score (IQR) was 50 (25–85) and 11 (3.2–28.2) for leisure activities. The professional sub-score was zero in 84.6% of participants. For domestic and leisure activities, the 10-50-90 percentiles gave the best-fit model. The splines were significant for the domestic (*p* = 0.0006), but not for the leisure activity scores (*p*-value = 0.063). The relation between cognitive disorders and professional activities vs. no activities was not significant (*p* = 0.388).

The classes obtained by the splines were [0–25], ]25 −60[, >=60 for domestic activities, and [0–15[, [15–50[, >=50 for leisure activities. For the domestic activities, the logistic regression model (Model 1) showed that the risk of cognitive disorders was lower for the second vs. the first class (Table 2) [OR (95% CI) equals to 0.27 (0.12–0.57)]. The results remained similar for the multi-adjusted model (Model 2): with OR (95% CI) equal to 0.31 (0.14–0.67). For the leisure and professional activities, the results were not significant, whatever the model used.

## Discussion

In this research, two different cut-offs of the MoCA were applied to assess cognitive decline and various dimensions of the PASE were used to assess the types of physical activity (professional, domestic and leisure). The study aimed at analyzing the relationship between different types of physical activity and incidence of cognitive disorders in community-living older adults. Using data from the FRéLE cohort, restricted cubic spline and logistic regression models, we found an inverse relationship between domestic sub-score and cognitive disorders defined by MoCA <18. The cubic spline curve showed a decreased risk with the leisure activities of the PASE, but the results were not significant.

Our results agree with two other studies which found the same trend with domestic activities in the risk of dementia: in a French cohort (1), which found that the risk of dementia was significantly and negatively associated with household/transportation activity level, but not with the leisure and sports activity sub-score, and a Swedish study (22), where physical activities as gardening, domestics activities or volunteer for older persons, was significantly associated with a lower risk of dementia 6.4 years later. These new results in the FRéLE cohort also showed a positive link between domestic activities and the incidence of cognitive disorders.

The strength of this study was the sample size of 1,271 participants in a longitudinal and observational cohort. This allowed for the analysis of an elderly population with a specific questionnaire. Moreover, it permitted to analyze different types of activities with quantitative information (three sub-scores) as could have been done in relation to mortality (23) and the place of residence (24). The use of continuous measures preserved all the information, improved the model and helped to determine the best levels of activity. Adjustment on covariates was important because of their relation with cognition. Age is a high risk factor of cognitive decline (25) and it has been shown that women had more risk (26, 27). Educational level is associated with cognition due to cognitive reserve (28). Multi-adjusted model included depression, cardiovascular diseases, arthritis, stroke or diabetes because they were significantly related to cognitive decline in the first logistic model and they are considered as risk factors (29).

Some limitations of our study is first of all, that at this aged range, the level of leisure and sports activities is low as a consequence of comorbidities. However, the effect of domestic activities persisted in the model adjusted for covariates. Also, the follow-up duration was relatively short. To limit the reverse causality issue, we excluded cognitive disorders at the first follow-up interview, but the small number of participants did not allow us to be conclusive. Finally, the PASE questionnaire generally evaluates self-reported PA, but this one gave information about the activity types. A cross-sectional study had presented the associations between PASE and MMSE (30), but due to the structure of the studies, it was not possible to clarify the causality direction. Furthermore, the type of activities in the PASE were not analyzed. In the Pianoro (31) Italian longitudinal study, no significant result was found between PASE and cognitive decline with a 7-year follow-up, but the three PASE scores were not analyzed separately. Another limitation of our study was the sample size. The ideal would have been to also assess the occurrence of severe cognitive disorders but our sample size did not permit it.

## Conclusion

This study showed the effect of domestic activities on the occurrence of moderate cognitive disorders. This component of physical activity is too often forgotten in physical activities, however they represent the majority of activities in older people. These findings will contribute to the ongoing debate on the beneficial effects of physical activity on cognition. Future researches would analyze the triad fragility/physical activity/cognition, so as to determined how fragility can influence before and in the same time, the relation between physical activity and cognition.

## Data Availability Statement

Public access to the database is closed. We received administrative permission to access and use these (https://www.maelstrom-research.org/mica/study/frele). The datasets used and/or analyzed during the current study are available from the corresponding author on reasonable request.

## Author Contributions

CD and BB participated in the conception and design of the study. CD conducted the analyses and wrote the first draft of the manuscript. CD, DH, LG, and BB participated in the interpretation of the data. IC, DH, LG, FB, FR, TC, NB, and BB critically reviewed the paper. BB and FB participated in the acquisition of the data. All authors contributed to the article and approved the submitted version.

## Conflict of Interest

The authors declare that the research was conducted in the absence of any commercial or financial relationships that could be construed as a potential conflict of interest.
